# Supplementary material for: Açaì (Euterpe oleracea) Extract Protects Human Erythrocytes from Age-Related Oxidative Stress
Source: Cells. 2022 Aug 3;11(15):2391. doi: 10.3390/cells11152391 (PMC9368007; doi:10.3390/cells11152391)
Supplement: Supplementary file 1 [file cells-11-02391-s001.zip › cells-1832016-supplementary.pdf]

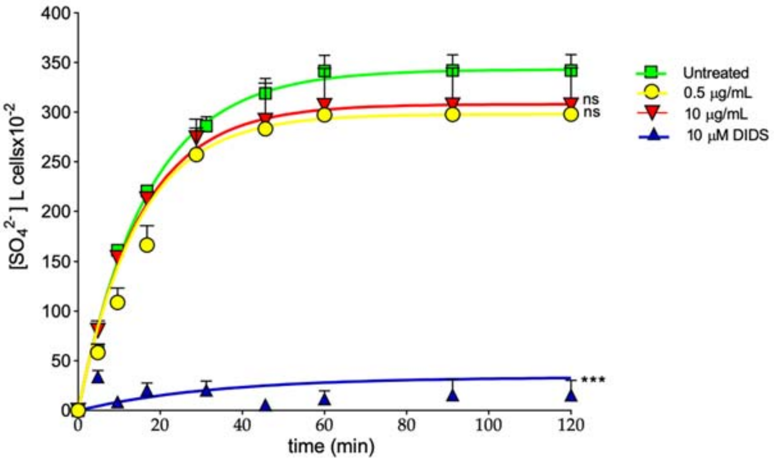

Figure S1. Time course of SO_4^{2-} uptake in erythrocytes left untreated (control) or treated with increasing concentrations (0.5 and 10 µg/mL) of freeze-dried Açaì extract (pre-incubation for 1 hour), or 10 µM DIDS. ns, not statistically significant versus control; ***, p<0.001 versus control, one way ANOVA followed by Bonferroni's post hoc test.
